# Open-Label placebo for the treatment of unipolar depression: Results from a randomized controlled trial

**DOI:** 10.1192/j.eurpsy.2023.1791

**Published:** 2023-07-19

**Authors:** U. Nitzan

**Affiliations:** Shalvata Mental Health Center and Tel-Aviv University, Tel-Aviv, Israel

## Abstract

**Introduction:**

The response to placebo is robust in studies of various antidepressant treatments. The strong placebo response, combined with the absence of side-effects, has prompted suggestions to use the ethically sound open-label placebo (OLP) as a treatment for depression.

**Objectives:**

The aim of the present study was to assess the efficacy of OLP in the setting of a randomized controlled trial for the treatment of unipolar depression.

**Methods:**

Thirty-eight patients (28 females, 73.7%) were randomized to either an eight-week treatment with OLP (n=18) or four week of treatment as usual (TAU) followed by four weeks of OLP (n=20). Clinical and socio-demographic measures were assessed at baseline, after four weeks, and at the end of the trial. Response to treatment was determined using the Quick Inventory of Depressive Symptomatology (QIDS SR-16).

**Results:**

There was an overall decrease in depression levels over time, *F*(2,35) = 3.98, *p* = .028). A significant *group* x *time* interaction was found only among non-geriatric patients (<65y) with an early onset of depression (<50y), *F*(2,22) = 3.89, *p* = .036]. Post-hoc tests indicated a significant decrease during the first four weeks, but only in the OLP group, *t*(11) = 2.29, *p* = .043.

**Table 1:** Demographic measures of OLP and TAU patients.

**Measures**	**OLP (*n* = 18)**	**TAU (*n* =20)**	**Statistical analyses**
Age (years) [Mean ± SD]	48.17 ± 16.86	51.65 ± 17.68	*t*(36) = -0.62, *p* = .539
Education level (years) [Mean ± SD]	15.61 ± 3.66	14.22 ± 2.62	*t*(34) = 1.31, *p* = .200
Gender (male/female) [no.]	4 / 14	6 / 14	*X*^2^(1) = 0.30, *p* = .587
Age of onset (years) [Mean ± SD]	34.19 ± 15.82	32.45 ± 17.72	*t*(36) = 0.32, *p*= .752
Number of depressive episodes (no.) [Mean ± SD]	2.58 ± 2.61	6.64 ± 9.16	*t*(21) = -1.47, *p* = .156
Number of hospitalizations (no.) [Mean ± SD]	0.12 ± 0.33	0.17 ± 0.38	*t*(33)= -0.40, *p* = .689

*Notes*

OLP = Open label placebo; SD = Standard deviation; TAU = Treatment as usual.

**Image 3:**

**Figure:**
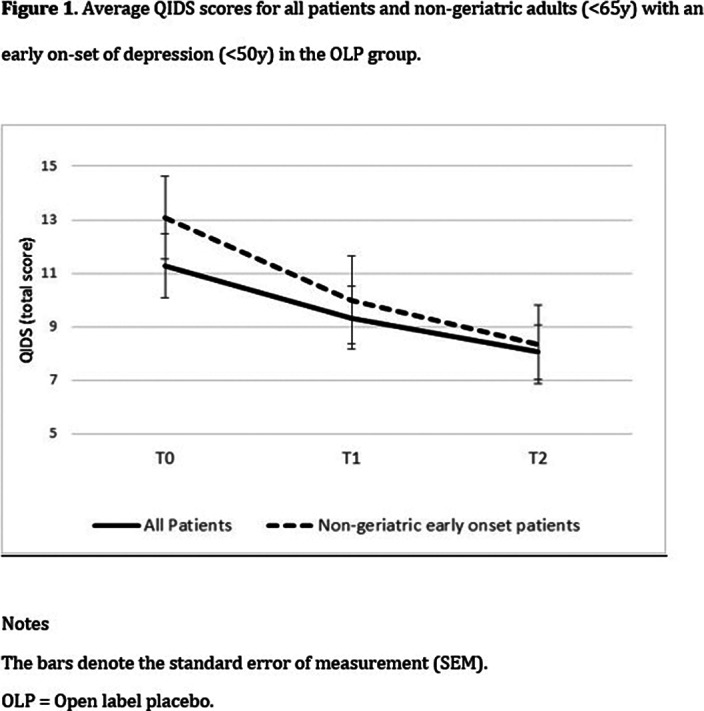

**Conclusions:**

Our findings support the possibility that OLP is an effective treatment for the relatively young population of patients suffering from depression. Additional studies are warranted in order to explore the use of open-label placebo in clinical work.

**Disclosure of Interest:**

None Declared

